# Determining the Association of the Dry Period Duration with Dystocia and Stillbirths in Dairy Cows by Considering Parity, Season, and Gestation Length

**DOI:** 10.3390/ani14101444

**Published:** 2024-05-13

**Authors:** Vigilijus Jukna, Edita Meškinytė, Ramūnas Antanatis, Algimantas Paulauskas, Vida Juozaitienė

**Affiliations:** 1 Center of Animal Production Research and Innovation, Agriculture Academy, Vytautas Magnus University, Universiteto St. 10A, Akademija, LT-53361 Kaunas, Lithuania; vigilijus.jukna@vdu.lt (V.J.); edita.meskinyte@vdu.lt (E.M.); 2 Large Animal Clinic, Veterinary Academy, Lithuanian University of Health Sciences, Tilžės Str. 18, LT-47181 Kaunas, Lithuania; 3 Agriculture Academy, Vytautas Magnus University, K. Donelaičio 58, LT-44248 Kaunas, Lithuania; algimantas.paulauskas@vdu.lt

**Keywords:** dairy cows, dry period, gestation period, calving difficulty, stillbirth, parity, season

## Abstract

**Simple Summary:**

Our research proposed a hypothesis suggesting that the duration of the dry period could potentially be correlated with the occurrences of dystocia and stillbirths, considering factors such as a cow’s parity, the year, the season, and the length of the gestation period. Moreover, it could have varying impacts on these crucial profitability indicators for dairy farms. The outcomes unveil notable connections between the incidence of dystocia and the length of the gestation period, calving season, and stillbirth. The key risk factors identified for stillbirths encompassed dystocia, parity, the calving season, the gestation period length, and the dry period length. Cows with dystocia had twice the odds of stillborn calves compared to those without dystocia. An increase in parity decreased the odds of stillborn calves. Cows calving from May to October had lower odds of dystocia and stillborn calves compared to those calving between November and April. A longer gestation period was associated with a higher probability of dystocia and calf mortality. Additionally, the analysis revealed that a prolonged dry period increased the odds of stillbirth. In summary, the findings of this study offer insights that may aid the dairy industry in making informed management decisions to mitigate the dystocia and stillbirth rates in cows.

**Abstract:**

This study aimed to assess the relationship between the length of the dry period (DL) and the risks of dystocia and stillbirths in Holstein cows. A total of 1072 healthy cows in lactations 1 and ≥2 were categorized based on the DL (mean: 58.07 ± 0.33 days) into three groups: short DL (<40 days; 13.0% of cows), medium DL (40–70 days; 60.1%), and long DL (>70 days; 26.9%). Dystocia occurred in 12.4% of cows, while stillbirths accounted for 4.1% of calves. The medium DL group had the highest number of unassisted calvings. The dystocia rates were 11.4% for cows with gestation (GA) ≤ 274 days; 6.0% for cows with GA = 275–282 days; and 19.9% for cows with GA ≥ 283 days (*p* < 0.001). In season 1 (November–April), dystocia occurred in 15.0% of cases compared to 8.8% in season 2 (May–October) (*p* < 0.001). The stillbirth rates ranged from 3.6% to 4.0% for cows with medium and long DLs; while those with a short DL had 5.8% (*p* < 0.05). The stillbirth rates were 5.2% for cows with GA ≤ 274 days; 3.2% for cows with GA = 275–282 days; and 5.1% for cows with GA ≥ 283 days (*p* < 0.001). Season 1 had a stillbirth rate of 4.8%, while season 2 had a stillbirth rate of 3.3% (*p* < 0.001). This research provides insights that could assist the dairy industry in making informed decisions to reduce the incidence rates of dystocia and stillbirths in cows.

## 1. Introduction

Before calving, cows require a dry period to facilitate mammary gland regeneration, involving both physical and physiological rejuvenation of the udder tissue [1]. Numerous studies have investigated optimizing the duration of the dry period and its impact on production-related factors [2,3,4,5]. Annen et al. [6], Kuhn et al. [7], and Sawa et al. [8] observed a consistent trend: a dry period shorter than 40 days or longer than 70–80 days resulted in significant decreases in milk production and the concentration of chemical components in the subsequent lactation.

The literature suggests that the duration of the dry period is associated with physiological changes in dairy cows, affecting aspects such as metabolism and health. High-yielding dairy cows undergo significant metabolic changes during the transition from the dry period in late gestation to the onset of abundant milk secretion in early lactation [9]. Prior to calving, homeorhetic changes induce significant alterations in the udder tissue through processes such as mammogenesis, lactogenesis, and galactopoiesis. These mechanisms promote the development of the alveolar system, enhance the synthetic capacity of udder cells, increase blood flow and nutrient intake, activate lactation, and sustain milk synthesis. The substantial nutrient demands for galactopoiesis are coordinated with changes in peripheral tissues, leading to mobilization or the formation of nutrients in these tissues to support milk production in the udder [10]. Steeneveld and colleagues suggest [11] that shortening the dry period (DP) has been proposed as a management strategy to improve energy balance in early lactation. However, when no dry period was applied to all cows in five commercial Dutch dairy herds, this resulted in a reduction in postpartum milk production (1–305 DIM) ranging from 3.2 to 9.1 kg/day, representing a reduction of 12% to 32%, respectively. Andersen with a group of scientists [12] concluded that for dairy cows that are expected to achieve peak milk yield exceeding 45 kg/d, omitting the dry period results in a notable reduction in the milk yield during the subsequent early lactation period. However, cows that managed without the dry period experienced fewer metabolic imbalances in early lactation due to the more favorable balance between nutrient intake and nutrient output in milk.

In light of this, it is essential to acknowledge that the relationship between the length of the dry period and animal welfare, including factors such as the frequency of difficult calvings and stillbirths, has often been overlooked in previous research. This aspect warrants further attention in future studies to ensure the well-being of both cows and their offspring.

The ease of calving and the survival of newborn calves are of significant economic importance in the dairy industry [13]. Difficulties during calving have been linked to decreased survival rates for both the cow and the calf, as well as diminished cow productivity, fertility, and lifespans. Calving complications can result in higher rates of neonatal calf mortality, reduced milk production, and an overall decline in the health of cows [14]. In recent decades, there has concurrently been a rise in average milk production per cow and a deterioration in health and fertility characteristics [15].

Stillbirth refers to the death of a calf occurring either just before, during, or within 24 to 48 h after parturition following a minimum gestation period of 260 days [16,17]. Stillbirths often result in compromised reproductive capabilities and decreased milk production and jeopardize the survival of the dam [18,19,20]. Studies have documented stillbirth rates ranging from 2.9% to 9.8% [21,22,23].

Dystocia and stillbirth can be the result of maternal factors, fetal factors, or a combination of both [24]. According to a prior scientific investigation, while dystocia is the leading cause of stillbirths, it only contributes to approximately 50% of such instances [25]. At the herd level, effectively managing dystocia relies on targeted sire selection, optimizing heifer growth, and strategically managing the dry period [24]. Dystocia scoring systems in the veterinary literature range from two-point [26] to seven-point [27] scales, with scores of two or higher typically indicating the need for assisted calving and scores of three or higher indicating a challenging calving process. A consistent finding across the existing research is that any form of calving assistance can lead to decreased subsequent fertility and productivity [28]. A study conducted by Probo and colleagues [29] in Italian Holstein cow herds revealed a correlation between the length of the dry period and calving ease. Cows with a dry period exceeding 70 days faced an increased risk of assisted calving compared to those with a dry period spanning from 44 to 70 days (odds ratio: 1.26; 95% CI: 1.15–1.38).

The duration of pregnancy can significantly influence cows’ calving outcomes, representing a critical factor in cattle breeding and performance [30]. Stillbirths have been linked to both prolonged and shortened gestation periods [23,31]. According to Nogalski and Piwczyński [30], the optimal gestation length falls within the range of 275–277 days, considering the occurrence of dystocia and stillbirth. However, Johanson and Berger [32] found that 282 days is the optimal gestation length for minimizing the risk of stillbirth. In the literature, we were unable to find reliable associations between the gestation period length from the minimum value of this indicator, which is used for assessing calving ease and calf mortality [16,17], and the dry period lengths of cows. However, research indicates that the risks associated with culling over the entire lactation period tend to slightly increase with both short and long dry period lengths (DPLs) compared to the reference DPL of 51 to 60 days [33].

Considering this, the primary objective of this study was to assess the impact of the duration of the dry period on the risks of dystocia and stillbirth in Holstein cows. This study hypothesized that the length of the dry period might be correlated with dystocia and stillbirths depending on a cow’s parity, the season, and the length of the gestation period. Furthermore, it could have varying effects on these critical profitability indicators for dairy farms.

## 2. Materials and Methods

### 2.1. Experimental Design, Animals, and Recording of Data

This study, conducted in compliance with the provisions of the Law on Animal Welfare and Protection of the Republic of Lithuania (study approval number PK016965), took place on a commercial dairy farm in the central region of Lithuania, which is located at 54°85′18.8″ N 23°16′99.6″ E. The investigation spanned from May 2019 to May 2023. The farm maintained around 1342 dairy cows, each with an average milk productivity of approximately 9000 kg per complete lactation.

Healthy cows free from clinical symptoms of any diseases were selected for this study, starting from at least the 200th day of pregnancy after their first to third calving. Holstein bulls used to inseminate these cows exhibited similar breeding values for both direct and maternal calving traits. This study involved the analysis of 3861 dry periods and calving records of 1072 healthy cows. Cows, when rated on a five-point scale, exhibited an average body condition score of 3.59 (with a standard error of ±0.16).

Based on their dry period lengths, the cows were classified into three groups: (1) short, with periods < 40 days, (2) medium, with periods between 40 and 70 days, and (3) long, with periods > 70 days.

Calving ease was assessed on a scale ranging from 1 to 5, where a score of 1 indicated no need for assistance, 2 signified a minor issue requiring some assistance, 3 indicated a moderate level of assistance, 4 denoted a significant amount of force or extreme difficulty during calving, and 5 signified extreme difficulty calving [34]. For statistical data analysis, a calving ease score of 1 was categorized as unassisted calving, while scores of 2 and 3 were classified as assisted calving, indicating slight to moderate difficulties. Scores of 4 and 5 were combined and categorized as difficult calving or dystocia.

A stillborn calf is defined as a calf that was born deceased or that passed away within the first 24 h after calving following a gestation period of at least 260 days. The average gestation length (GL) for cows was recorded as 278 ± 9.2 days. The duration of pregnancy was categorized into three classes: (1) short (GL ≤ 274 days), (2) optimal (GL = 275–282 days), and (3) long (GL ≥ 283 days). Notably, based on the research by Nogalski and Piwczyński [30] and Johanson and Berger [32], GL class 2 was identified as the optimal duration of pregnancy.

The dry period was analyzed in cows from the end of the first lactation (cows were categorized by lactation as follows: lactation 1 and lactation ≥2), and its relationship with calving ease and calf stillbirth rates was assessed starting from the second calving (parity: 2 and ≥3).

Taking into account the sample structure and temperature conditions, the cows were divided based on the season of calving: season 1 (November–April) and season 2 (May–October).

When evaluating the outcomes of cow calving in relation to the duration of the cows’ dry periods, the analysis excluded data on twins. Over the study period, cows gave birth to 2046 heifers (52.99%) and 1815 bulls (47.01%), but the primary data analysis did not indicate the necessity of analyzing data categorized by calf sex.

The cows were kept in the barn for the entire year and followed a zero-grazing system. The total mixed ration (TMR) in all farms was balanced to fit the energy and nutrient requirements of a 550–650 kg Holstein cow (Table 1).

### 2.2. Statistical Analysis of Data

A statistical analysis of these study data was performed using SPSS 25.0 software (IBM Corp. Released 2017. IBM SPSS Statistics for Windows, Version 25.0. Armonk, NY, USA: IBM Corp).

Pearson’s chi-squared test was used to determine if there was a statistically significant difference between category frequencies in the groups.

The multivariable binary logistic regression method was employed to evaluate the risk of stillborn calves in cows by considering factors such as the duration of the dry period (categorized into three groups), the gestation period length (categorized into three groups), parity (categorized into two groups), season (categorized into two-year seasons), and calving ease (assessing the presence or absence of dystocia). Similarly, the risk of dystocia was examined using logistic regression and by incorporating the same factors and the incidence of stillbirth in calves (defined as a live calf born or one that died within 24 h after delivery). Only significant explanatory variables were included in the final statistical models utilizing a backward stepwise logistic model to exclude non-essential variables based on the significance of the Wald test.

A probability of less than 0.05 was considered statistically significant (*p* < 0.05) for all tests.

## 3. Results

### 3.1. Distribution of Cows Based on Dry and Gestation Periods

The analysis revealed that the average dry period of cows during the four years of this study was consistent at 58.07 ± 0.33 days (ranging from 57.12 to 59.00 days). Cows with a short dry period (*n* = 503) accounted for 13.0% of the total, those with a medium period (*n* = 2321) constituted 60.1%, and those with a long period (*n* = 1037) represented 26.9%. The difference in the number of cows with a medium dry period varied insignificantly by year (0.98–1.8%).

**Figure 1:**
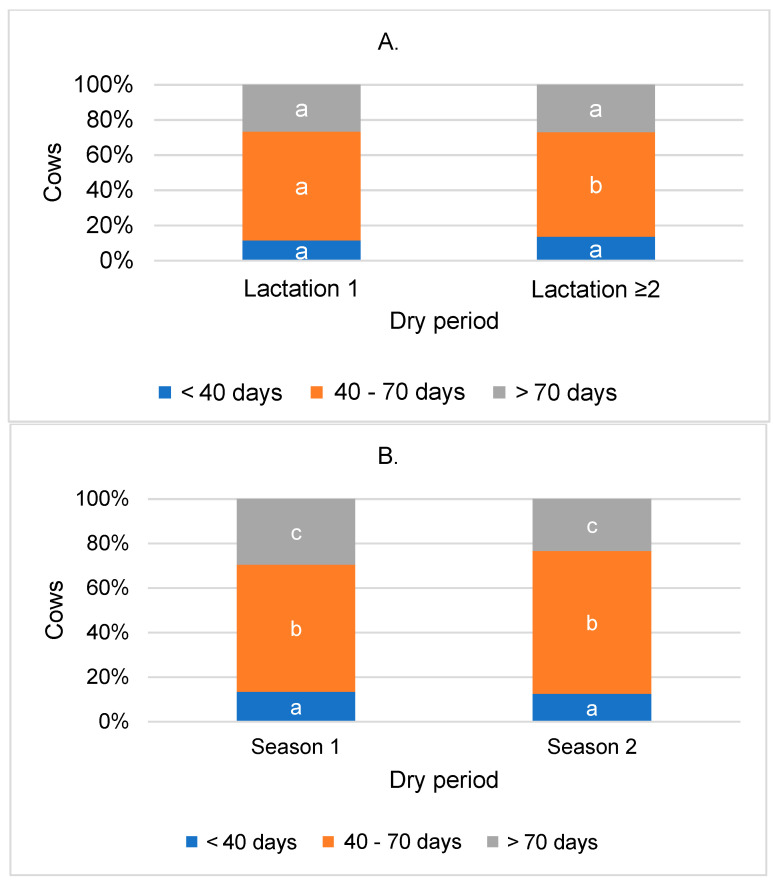
Distribution of cows by dry period length, categorized by lactation (**A**) and season (**B**). Season 1: November–April; season 2: May–October. Statistical significance exists between dry period groups categorized by lactation or season, designated with different letters; *p* < 0.05.

The dry period in cows exhibited slight variations in lactation number. After the first lactation, there were 2.0% fewer cows in the sample with a short dry period and 2.3% more cows with a medium dry period compared to multiparous cows (Figure 1A).

This study shows that there were 7% more cows with a medium dry period and, correspondingly, 7% fewer cows with a long dry period during the warmer season of the year (May–October) compared to the colder season (November–April) (*p* < 0.001) (Figure 1B).

**Figure 2:**
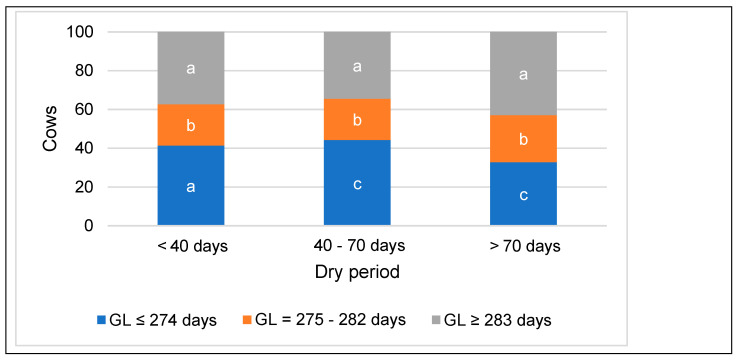
Distribution of cows based on gestation period (GL), categorized by dry period. Statistical significance exists between dry period groups categorized by gestation period, designated with different letters; *p* < 0.05.

According to the gestation period, the cows were distributed as follows: short—40.8% of cows; optimal—22.1%; and long—37.1%. An increase in the duration of the dry period in cows was associated with an increase in the gestation period (Figure 2). Most cows with a gestation period ≥ 283 days were found in the group with a long dry period (5–8% more than in other groups; *p* < 0.001).

### 3.2. Distribution of Cows Categorized by Their Calving Ease Score

According to the calving ease score, the cow sample was distributed as follows: a score of 1 (*n* = 1688) accounted for 43.7% of cows, a score of 2 (*n* = 1255) represented 32.5% of cows, a score of 3 (*n* = 441) made up 11.4% of cows, and scores of 4 and 5, representing dystocia (*n* = 477), comprised 12.4% of cows. The cases of dystocia slightly decreased over the four years (from 13.1% to 12.0%) in the study cattle herd.

**Figure 3:**
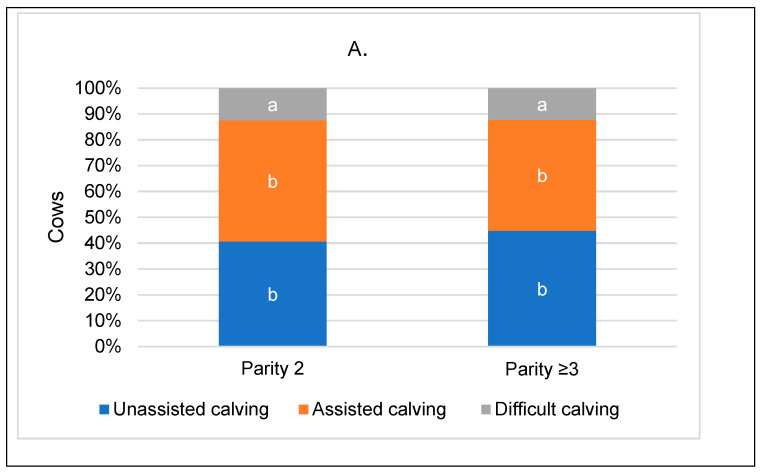

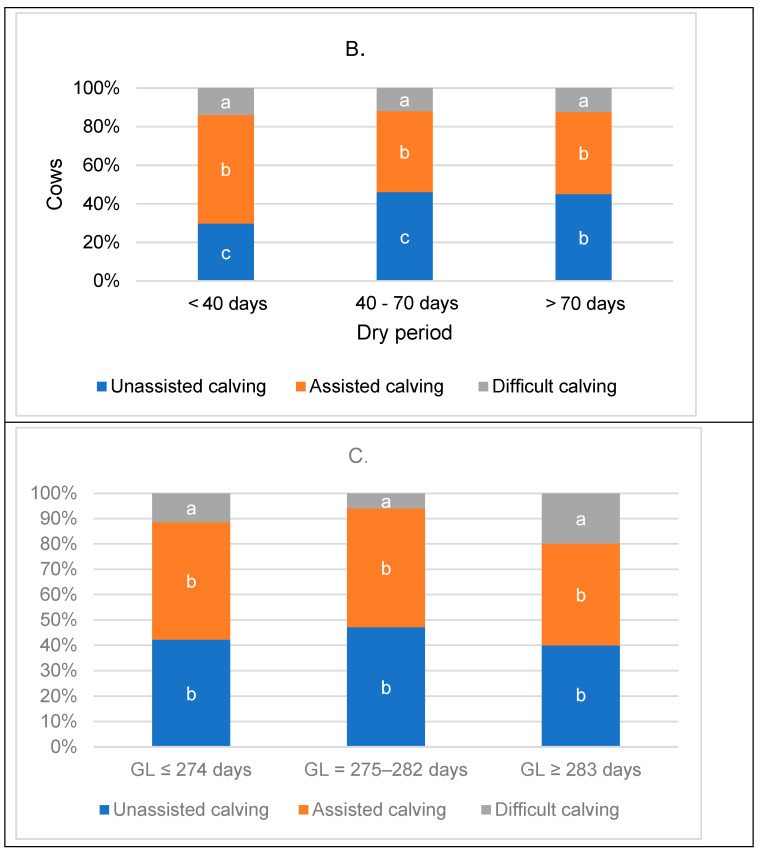
Distribution of cows by calving assessment, categorized by parity (**A**), dry period length (**B**) and gestation period (**C**). Statistical significance exists between calving ease groups categorized by parity; dry period and gestation period designated with different letters; *p* < 0.05.

Unassisted calving occurred in cows of parity 2 in 4.2% more cases compared to parity ≥ 3. On the other hand, the incidence of assisted calving was 4.0% higher in cows of parity 2. The occurrence of difficult calving was at a similar level in both groups of cows (Figure 3A).

Difficult calving occurred in cows with a short dry period in 1.9% more cases (*p* < 0.001) and in cows with a long dry period in 0.4% more cases compared to the group with a medium dry period (Figure 3B). The highest number of cows with unassisted calving was in the medium dry period group, which was 16.3% higher than in the short dry period group (*p* < 0.001) and 1.1% higher than in the long dry period group. According to the chi-squared test data for all groups of cows, we can conclude that the dry period of the cows was significantly associated with the calving ease of the cows (*p* < 0.001).

Dystocia cases represented 11.4% of cows with a GL ≤ 274 days, 6.0% of cows with a GL = 275–282 days, and 19.9% of cows with a GL ≥ 283 days (*p* < 0.001). Cases of unassisted calvings were found to be 4.90% and 7.22% lower (*p* < 0.001) in the groups with short and long gestation periods, respectively, compared to the group of cows with optimal gestation periods (Figure 3C). In season 1, we observed an estimated 15.0% prevalence of dystocia cases in cows, whereas in season 2, the prevalence was 8.8% (*p* < 0.001).

### 3.3. The Distribution of Cows Based on the Stillbirth Rates of Their Calves

We found a stillbirth rate of 4.1% among all calf births, and this indicator consistently decreased from 4.3% to 3.9% over the study period. The percentage of stillbirths in cows of parity 2 (5.3%) was 1.60% higher than that in multiparous cows (*p* < 0.05). The stillbirth rate of calves remained relatively consistent throughout the years of the experiment, ranging from 3.9% to 4.2%.

**Figure 4:**
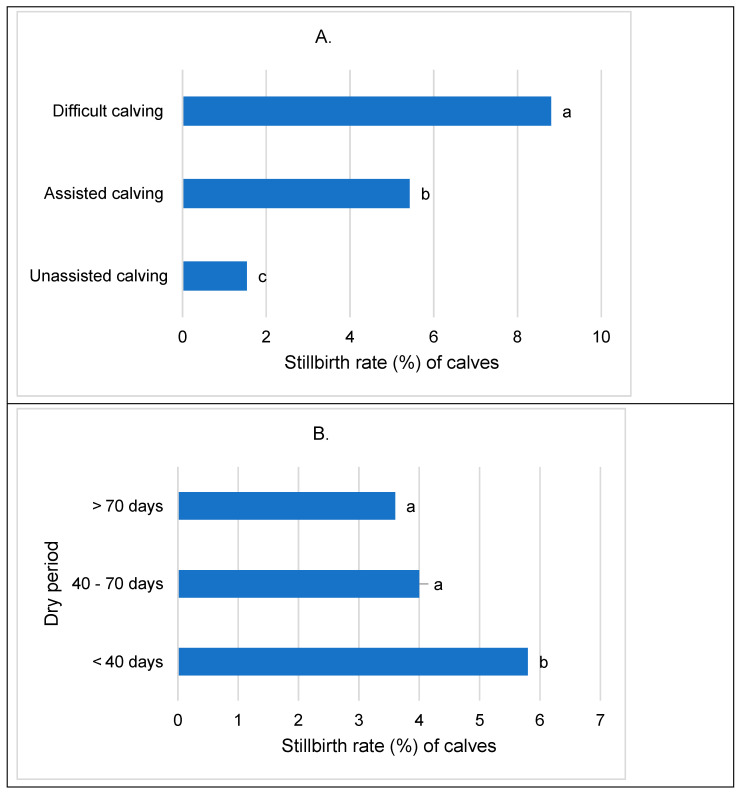

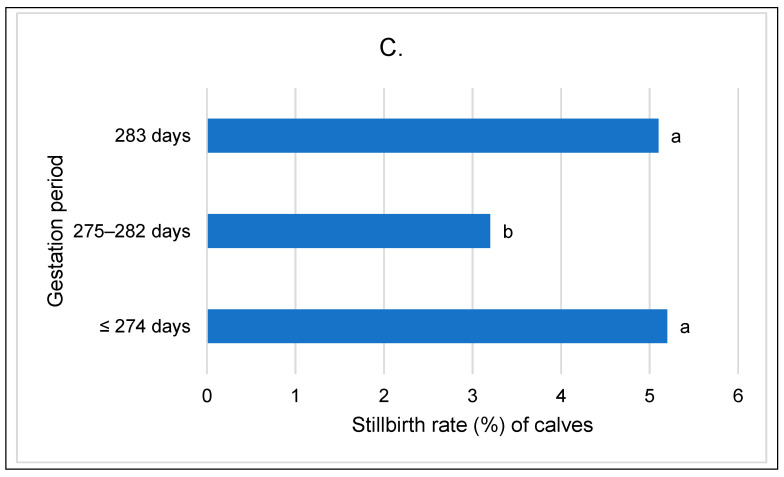
Stillbirth rate (%) of calves based on calving assessment of cows (**A**), dry period length (**B**) and gestation period (**C**). Statistical significance exists between groups designated with different letters; *p* < 0.05.

The stillbirth rate of calves in cows with unassisted calving was 1.5%, which is 3.9% lower (*p* < 0.001) than that in cows with assisted calving and 7.3% lower than that in cows with difficult calving (Figure 4A). An increase in the calving assessment score was associated with a risk of stillbirth that was 1.8 times higher among calf births (*p* < 0.001).

Stillbirths among calves decreased as the dry period shortened (Figure 4B). Specifically, the stillbirth rate ranged from 3.6% to 4.0% in cows with a medium to long dry period, whereas it was 5.8% in cows with a short dry period (*p* < 0.05).

The stillbirth rate of calves showed variability across gestation lengths: it was 5.2% for cows with a gestation length (GL) of ≤274 days, 3.2% for cows with a GL of 275–282 days, and 5.1% for cows with a GL of ≥283 days. It was found that the number of stillbirths in cows with short and long gestation periods was similar and significantly higher (*p* < 0.001) than the rate in cows with an optimal gestation period (Figure 4C).

In season 1, we observed an estimated 4.8% prevalence of stillbirths in calves among cows, while in season 2, the prevalence was 3.3% (*p* < 0.001).

### 3.4. Factors Influencing the Stillbirth Rate of Calves and the Incidence of Dystocia in Cows

Cows with stillbirths had 2.233 times higher odds of dystocia compared to those with normal calf survival (95% CI = 1.630–2.797, *p* < 0.001). Long gestation periods showed a 1.434-fold increase (95% CI = 1.167–1.603, *p* < 0.001) in dystocia odds, while short gestation periods had a slightly lower 1.360-fold increase (95% CI = 1.511–1.702, *p* < 0.001) compared to the optimal gestation period. Additionally, cows calving during the May–October period had odds of dystocia that were 0.618 times smaller (95% CI = 0.483–0.812, *p* < 0.001) compared to those calving between November and April (Table 2, model 1).

When examining factors linked to calf mortality (Table 2 and model 2), a multivariable logistic regression analysis revealed that cows with dystocia had 2.144 times higher odds (95% OR = 1.522–2.851, *p* < 0.001) of stillborn calves compared to cows without dystocia. Conversely, an increase in parity decreased the odds (by 0.701 times, 95% CI = 0.470–0.972, *p* < 0.001) of stillborn calves. Similarly, cows calving during the May–October period showed 0.352 times smaller odds (95% CI = 0.207–0.483, *p* < 0.001) compared to those calving between November and April. A long dry period was associated with a 1.773-fold increase in the odds of stillbirth in calves (95% CI = 1.401–2.002, *p* = 0.011), while a short period did not exhibit significant changes in odds compared to a medium dry period. Both short and long gestation periods were associated with significantly higher odds of stillbirth in calves (1.206–1.601 times) compared to the optimal gestation period.

## 4. Discussion

The present study postulated a hypothesis indicating a potential association between the duration of the dry period and the occurrence of dystocia and stillbirths. This association was anticipated to be influenced by factors such as the cow’s lactation number, year, season, the length of the gestation period, and the sexes of calves.

The distribution of cows according to the calving ease score was as follows: 1 (43.7% of cows), 2 (32.5%), 3 (11.4%), and 4–5 (12.4%). In this study, scores of 4–5 were classified as difficult calving or dystocia. Dystocia is frequently described as a substantial level of difficulty during calving [35]. The occurrence of dystocia in the United States varies between 5.0% and 6.6% in multiparous cows (1.9–2.0 times lower than in primiparous cows from 1985 to 1996) [36]. In Iranian Holstein dairy cows, the documented prevalence of dystocia is 8.2% [37]. Wall et al. [38] observed that 16.0% of cows in the United Kingdom require assistance during calving, and the worldwide prevalence of dystocia is estimated to range from 1.5% to 22.0% [35].

This study revealed a 4.1% mortality rate among calves within the initial 24 h post-birth. A rise in the calving score correlated with a 1.8-fold increase in the risk of stillbirth among calves (*p* < 0.001). The highest stillbirth rate (8.8%) among calves occurred in cases of dystocia. In parallel, Bahrami-Yekdangi et al. [39] observed an average dystocia incidence of 14.7% and a stillbirth incidence of 4.3%. Their findings align with our study, emphasizing a significant association between stillbirths and dystocia.

We observed 1.5–1.7-fold increases in cow dystocia and calf stillbirth cases during season 1 (November–April) compared to season 2 (May–October). Johanson and Berger [32] found that calves born in the winter are 36% more likely to die in the first 48 h than those born in the summer.

The lowest frequency of dystocia (6.0%) and the lowest stillbirth rate of calves (3.2%) were documented in cows with a gestation period ranging from 275 to 282 days. Such pregnancy length was observed in 22.1% of the cows in the study sample. Based on the research by Nogalski and Piwczyński [30] and Johanson and Berger [32], such length of pregnancy was identified as optimal. Meyer et al. [36] noted that short gestation periods pose the most challenges. In our study, the cows’ gestation periods were documented to be a minimum of 260 days when assessing their calving outcomes.

The highest stillbirth rate among calves, along with an increased incidence of dystocia cases in cows, was observed in cows with both short and long gestation periods compared to those with an optimal gestation period.

Extended dry periods are often linked to both shorter lactation lengths and gestation periods. According to Nogalski and Piwczyński [30], there exists a direct correlation between the gestation length and calf birth weight, indicating that longer gestation periods result in heavier calves, potentially leading to challenging calving situations. Prolonged dry periods, coupled with shorter lactation periods, may increase the risk of dystocia due to fetal oversize.

Considering the duration of the dry period, which averaged 58.07 ± 0.33 days, the cows were classified into three distinct groups: those with a dry period lasting less than 40 days, constituting 13.0% of the total cow sample; cows with a dry period ranging between 40 and 70 days, representing the majority at 60.1%; and cows experiencing a dry period exceeding 70 days, comprising 26.9% of the total cow sample. The traditional practice of 305 days of lactation and a 51- to 60-day dry period has been in use since World War II [40]. An analysis of the literature revealed that a dry period shorter than 40 days or longer than 70–80 days was associated with a significant reduction in the milk production of cows in the subsequent lactation [6,7,8]. Collier et al. [5] observed that the dry period is necessary for an optimal milk yield in the next lactation by facilitating cell turnover in the bovine mammary gland. With increasing milk production per cow, transitioning cows from the non-lactating state to peak milk yield has become challenging, prompting new studies on dry period requirements. These studies highlight a clear parity effect, with first-parity animals requiring a 60-day dry period. In contrast, later parities show no negative impact when somatotropin (ST) is used to maintain milk yields. Shortened dry periods in first-parity animals were associated with reduced mammary cell turnover, increased senescent cells, and decreased functionality of lactating alveolar mammary cells postpartum. The use of ST and increased milking frequency postpartum mitigated the impact of shortened dry periods.

Nevertheless, determining an optimal dry period length is a complex process influenced by factors such as parity, herd size, and milk production levels [41]. It is crucial to quantify the potential impact of this period on subsequent lactation performance to make informed decisions about the optimal dry period length in dairy cows [2,6]. Reducing the dry period length between lactations in dairy cows has been an active research focus for several years [3,4,5,41,42]. The emphasis of research on this matter has primarily centered on the productivity of cows. However, we believe this focus is inadequate as it overlooks crucial aspects such as animal welfare, the calving ease of cows, and the survival rates of calves. Neamt et al. [43] discovered that the incidence of dystocia increases as the length of the dry period extends, reaching critical levels for intervals exceeding 80 days. Calves’ mortality rates were found to be comparable for dry periods shorter than 80 days but were considered critical in cows with dry periods exceeding 80 days.

In summary, cows experiencing stillbirths had significantly higher odds of dystocia compared to those with normal calf survival. Long gestation periods were associated with a notable increase in dystocia odds, while short gestation periods showed a slightly lower increase compared to the optimal gestation period. Moreover, cows calving during the May–October period had lower odds of dystocia compared to those calving between November and April.

When analyzing factors related to calf mortality, multivariable logistic regression found that cows with dystocia had 2.144 times higher odds of stillborn calves compared to those without dystocia. Conversely, an increase in parity reduced the odds of stillborn calves. Cows calving during the May–October period had lower odds compared to those calving between November and April. A long dry period increased the odds of stillbirth, while short and long gestation periods were associated with higher odds compared to the optimal gestation period.

## 5. Conclusions

After investigating the hypothesis that the length of the dry period might be correlated with dystocia and stillbirths depending on factors such as cow parity, the season, and the length of the gestation period, and determining that it could have varying effects on these critical profitability indicators for dairy farms, we found that the key risk factors for stillbirths included dystocia, parity, the calving season, the gestation period length, and the dry period length. Cows with dystocia had twice the odds of stillborn calves compared to those without dystocia. An increase in parity reduced the odds of stillborn calves. Cows calving during the May–October period had lower odds of dystocia and stillborn calves compared to those calving between November and April. An increase in the gestation period was associated with higher probabilities of dystocia and calf mortality. The analysis also showed that a prolonged dry period increased the odds of stillbirth.

## Figures and Tables

**Table 1:** The components of the total mixed ration for dry cows.

Components	Daily Feeding for Each Animal (kg/cow/day)
Rapeseed 36%	1.2
Grass silage (27% DM)	8
Maize silage (27% DM)	1.2
Wheat straws	7.5
Water	4.3
Dry cow mineral and vitamins	0.250

DM—dry matter.

**Table 2:** An analysis of the risks of stillbirth in calves and dystocia in cows.

Explanatory Variables	OR	95% CI		*p*
		Min	Max	
Model 1: Dystocia in cows				
Gestation period				<0.001
-Short	1.360	1.511	1.702	<0.001
-Long	1.434	1.167	1.603	<0.001
Season	0.618	0.430	0.812	<0.001
Stillbirth of calves	2.233	1.630	2.797	<0.001
Model 2: Stillbirth in calves				
Dystocia	2.144	1.522	2.851	<0.001
Gestation period:				0.020
-Short	1.206	1.008	1.497	0.050
-Long	1.601	1.309	1.974	0.010
Season	0.352	0.207	0.483	<0.001
Parity	0.701	0.470	0.972	0.043
Dry period				0.049
-Short	1.398	1.002	1.763	0.089
-Long	1.773	1.401	2.002	0.011

OR—odds ratio; 95% CI—95% confidence interval for odds ratio. Reference categories for explanatory variables: dystocia—no dystocia; stillbirth of calves—calf was born alive; gestation period—optimal gestation length; dry period—medium dry period; season—season 1 (November–April); parity—parity = 2 and parity ≥ 3. *p*—*p*-value (considered statistically significant when *p*-value < 0.05).

## Data Availability

The data presented in this study are available within the article.
